# Assessment of pricing, availability, and affordability of essential medicines in a resource-limited setting

**DOI:** 10.1080/20523211.2026.2701916

**Published:** 2026-07-30

**Authors:** Faiza Inayat, Nasir Mahmood, Bashir Ahmad, Muhammad Sheeraz Ahmad

**Affiliations:** a Department of Economics and agricultural economics, PMAS Arid Agriculture University Rawalpindi, Rawalpindi, Pakistan; b University Institute of Biochemistry and Biotechnology (UIBB), PMAS Arid Agriculture University Rawalpindi, Rawalpindi, Pakistan

**Keywords:** Essential medicines, affordability, resource limited, pricing, availability, low resource setting

## Abstract

**Background:**

Access to essential medicine is a worldwide issue, driven by high prices, low affordability and limited availability. Under funded health systems, weak local production, poor supply chain governance, and discriminatory policies are the main hurdles. Low and middle-income countries are continuously making efforts to improve healthcare services. To evaluate the extent to which the intended objectives have been met, the present study undertakes an investigation into the pricing, availability, and affordability of selected essential medicines in Pakistan.

**Methods:**

A survey of 50 essential medicines, including 14 from the global core list and 36 selected from the national essential medicine list, was carried out from November 2025 to February 2026, using an adaptation of WHO/HAI methodology in five geographical areas of Pakistan. Data were acquired from 9 pharmacies of government hospitals and 18 retail pharmacies of the private sector. Prices were evaluated on the basis of the Median Price Ratio (MPR); affordability was determined by estimating how many days a minimum salaried employee of the government must spend to buy the prescribed regimen.

**Results:**

In the public sector mean availability was 12.65% (IB), 36.2% (LPG) and in private sector it was 45.7% (IB) and 55.2% (LPG). In private retail pharmacies, 13.61% were stocked with oncology medicines. Median price ratios in the private sector varied between 0.11–6.85 for LPG and 0.17–23.37 for IB. Median price ratio of 12 medicines (out of 36 calculated for MPR) was approximately similar between LPG and IB. However, prices for cardiovascular and antidiabetic medicines increased.

**Conclusion:**

Availability of LPGs has improved in the private sector. Median price ratios have decreased over time, but medicines are still unaffordable, especially for diabetes, viral, fungal and bacterial infectious diseases.

## Introduction

Access to healthcare has always been a significant social, political, and economic issue across the globe. This issue is prominently recognised under Sustainable Development Goal 3 of the 2030 Agenda. ‘Ensure Healthy Lives and Promote Wellbeing of all at all ages’ (United Nations Human Rights Council, 2025). These goals cannot be achieved without a strong health care infrastructure and fair access to medicines. Despite this global recognition, many in the global population continue to lack access to affordable and safe healthcare services. According to the United Nations Human Rights Commission's 3rd report on access to medicine, ‘2 billion people globally lack access to essential medicines’ (United Nations Human Rights Council, 2025); expanding availability could prevent millions of deaths each year (World Health Organization, 2023). Although core priorities for essential medicines such as funding , affordability, safety, proper use, and innovation were identified a decade ago (Wirtz et al., 2017), meaningful progress is still lacking. Wider global concerns, including the tension between intellectual property and human rights, as well as weak health systems and unequal access, remain unresolved. From a strategic perspective, embedding pharmaceutical policy within the framework of universal health coverage can create opportunities for more integrated health outcomes (Bigdeli et al., 2015).

Pakistan, a South Asian, lower-middle-income country, has gone through major changes in population dynamics and health trends throughout the previous decades. Some of these are rapid population growth, urbanisation, and improvements in life expectancy, along with the disease burden shifting from being dominated by communicable disease to increasingly centre on non-communicable disease (Kazmi et al., 2022). According to the World Bank report of 2023, just 2.52% of total GDP is granted to healthcare, with per capita spending amounting to just $30.4, out of which 52.93% is out of pocket (World Bank, 2025). The reliance on the private sector has significant financial implications for households. Pakistan integrates a dual health care system, including public and private health sectors that function in parallel. The system of public health care comprises rural health centres, basic health units, hospitals at Tehsil headquarters, and District Headquarters hospitals (Nishtar et al., 2013). Under this system, it’s a principle to provide free consultation and medicines, but persistent challenges related to underfunding, procurement inefficiencies, weak supply chain management, governance constraints, as well as political instability have led to repeated shortages and restricted access to essential medicines within public sector facilities. The patients who are admitted to public hospitals after being checked by consultants free of charge, often buy medicines from private pharmacies (Zaidi et al., 2013).

The prices of essential medicines are regulated in Pakistan, unlike many other countries of the world, such as Malaysia, that has no control over prices (Babar et al., 2007), and Nepal, which has no proper policy yet (Babar et al., 2024). Pakistan established a drug regulatory authority in 2012. However, enforcement gaps and rising supply chain costs have contributed to large price variations and scarcities. Consequently, households largely rely on out-of-pocket payments for the purchase of medicines. Healthcare resources are distributed unfairly, creating a serious problem (Khan et al., 2023). Access to affordable medicine, price regulation, and effective control over prices and market sustainability have become a permanent challenge. The system is facing structural challenges due to complex interplay of drug regulation and public access (Ali et al., 2021). High medicine prices remain a major barrier, especially for chronic and lifesaving treatments, disproportionately affecting low-income households (Zaidi et al., 2013). Research indicates that frameworks of pricing in low- and middle-income countries, such as Pakistan, often face difficulties in reconciling the commercial priorities of the pharmaceutical industry with the imperative of ensuring patient affordability (Babar, 2022; Babar, Jamshed, et al., 2013).

Pakistan has periodically revised its Drug Pricing policy, most notably in 2012, 2015, and 2018 in order to address affordability concerns by linking annual price adjustments to inflationary indicators; it also allowed exceptional increases for cost-driven cases (Rafique et al., 2023). Despite these efforts, the debate continues regarding the effectiveness, transparency, and public interest orientation of pharmaceutical price regulation in ensuring sustained access to affordable medicines. Pakistan needs to observe global evidence-based pricing strategies (World Health Organization, 2020) to update and implement one or a mix according to the situation to increase fair pricing and transparency (Loh et al., 2026). However, to achieve these goals, capacity building, cross-sectional collaboration, and sustained political commitment are required, as improving pharmacy practice and policy in LMICs is not merely a technical exercise but an ongoing process of system transformation deeply intertwined with social justice and governance reforms (Babar, 2021, 2025).

In private sector medicine, prices vary due to differences in manufacturers of generic substitutes, mark ups across the supply chain, and limited price transparency (Abdullah et al., 2024). Although Pakistan has a national mechanism for regulating medicine prices, its effectiveness has been a subject of ongoing debate. Over the past decade, Pakistan’s pharmaceutical sector has undergone significant regulatory shifts. In 2015, the government linked the essential medicine prices to the consumer price index (CPI) and allowed annual adjustments of approximately 7–10 per cent (Lee et al., 2017). However, this mechanism was suspended due to the manufacturing industry's misuse of this permission, and the government froze price increases (Lee et al., 2017). Due to price controls, financial strain on multinational corporations (MNCs) operating in the country increased, as rising production costs, local competitors, and political instability compelled many to leave (Khan, 2024). The situation changed in 2024, when the federal cabinet deregulated non-essential medicines, letting market forces set prices (*The Express Tribune*, 2024). A comprehensive evaluation of the overall situation is required to monitor and analyse it, but data and policy research are not carried out at a sufficient level (Babar, 2022).

## Rationale of the study

Previous studies in Pakistan were conducted based on data from nearly a decade ago, covering one district of one province (Saeed et al., 2019), followed by a study in Gujranwala district of the Punjab province (Shahzad et al., 2022). Another study covered just the Balochistan province (Bibi et al., 2022). All these studies are limited in scope to a single province or district. The last nationwide survey was carried out twenty-two years ago under a project of WHO (Kiani et al., 2006). Since then, pharmaceutical pricing has undergone many changes and faced significant challenges. In light of these developments, a comprehensive situation analysis is required to evaluate the current situation.

The principal objective of the present investigation is to assess the pricing, affordability, and availability of medicines in Pakistan, and to evaluate whether medicine prices are consistent across the country. Furthermore, the study aims to evaluate the impact of disparities in the prices of the originator brands compared to their generic equivalents on access to medicines.

## Methodology

### Research framework and setting

A cross-sectional survey was undertaken from November 2025 to February 2026 across Pakistan. Data on medicine pricing were collected from private sector retail pharmacies to make a comparison with international reference prices and to assess affordability. Data on availability were collected from private and public sector pharmacies to provide a comparative assessment of medicine access.

### Selection of medicines

An adjusted version of WHO/Health Action International methodology is followed to survey a total of 50 medicines, out of which 14 belong to the global core list; this list is finalised by WHO according to the global disease burden (World Health Organization, 2008), and 36 supplementary medicines were added according to local disease burden and physicians' prescriptions in Pakistan (Abdullah et al., 2024), keeping in view the pattern of medication. All the medicines are part of Pakistan’s National Essential Medicine List 2025 (NEML, 2025). It was also considered that most of the medicines have an international reference price according to the MSH guide (Management Sciences for Health (MSH), 2015).

### Selection of sampling areas for survey

A systematic random sampling technique was used to conduct a nationwide survey, taking Islamabad as the centre and two major cities from each province: Lahore and Rawalpindi from Punjab, Karachi and Kashmore from Sindh, Peshawar and Mansehra from Khyber Pakhtunkhwa, and Quetta and Naseerabad from Balochistan. In provincial headquarters, one major tertiary care public hospital and 2 pharmacies situated within a 10 km area were selected for the survey. In the main districts, one DHQ was selected to record availability and 2 private retail pharmacies were chosen to collect data on pricing and availability. The principal investigator arranged training sessions for the data-collecting personnel. The data for Innovator brand (IB) and lowest priced generic (LPG) were collected from private pharmacies, and availability was confirmed by physical presence during the survey. A retail pharmacy with less than 50 per cent of medicines was not selected for the survey as per the guidelines of WHO/HAI.

### Collection of data and analyses

Innovator brand and lowest-priced generic prices at each facility were documented. The price per unit was collected and then entered in the workbook of international medicine prices (World Health Organization, 2008). To ensure data accuracy, prices were entered twice in the workbook, and automated validation checks were applied. The software computed the Median Price Ratio (MPR) only when the medicine was available in at least four facilities. International reference prices were sourced from the 2015 International Drug Price Indicator Guide published by Management Sciences for Health (MSH) (Management Sciences for Health (MSH), 2015). The supplier median prices of 36 surveyed medicines were obtained, and the exchange rate from the Forex.pk currency converter on March 31, 2026, was used to convert prices into US dollars (Forex.PK, 2026). These values were then adjusted for inflation to ensure comparability. The International Reference Prices (IRPs) represent the median of recent procurement or tender prices, primarily from non-profit suppliers. The median supplier price of 36 surveyed medicines was converted into US dollars and then adjusted for inflation. An MPR of > 2 was considered the threshold for overpriced medicines, consistent with criteria used in prior studies conducted in Pakistan using the same methodology. (Kiani et al., 2006; Saeed et al., 2019).

Affordability was calculated using the WHO/HAI standard methodology to measure medicine pricing and availability. Affordability was calculated using the per-day wage of the lowest-paid unskilled government worker. In Pakistan, the lowest pay is the minimum wage, set at 1233 Pakistan Rupee (PKR) by the federal government in 2025 (Business Recorder, 2025). Hence, the minimum wage was used in Pakistan and in this paper.

### Research ethics

The Research Ethics and Conflict of Interest Committee, PMAS-Arid Agriculture University, Rawalpindi, under reference letter No. PMAS-AAUR/IEC/266 dated 23-08-2025 granted approval for this research. Additionally, a formal letter was obtained from the Department of Economics to be submitted to the Medical Superintendent of public hospitals, seeking permission to conduct data collection at each public health facility.

## Results

### Median price ratios (private sector)

**Figure 1.**
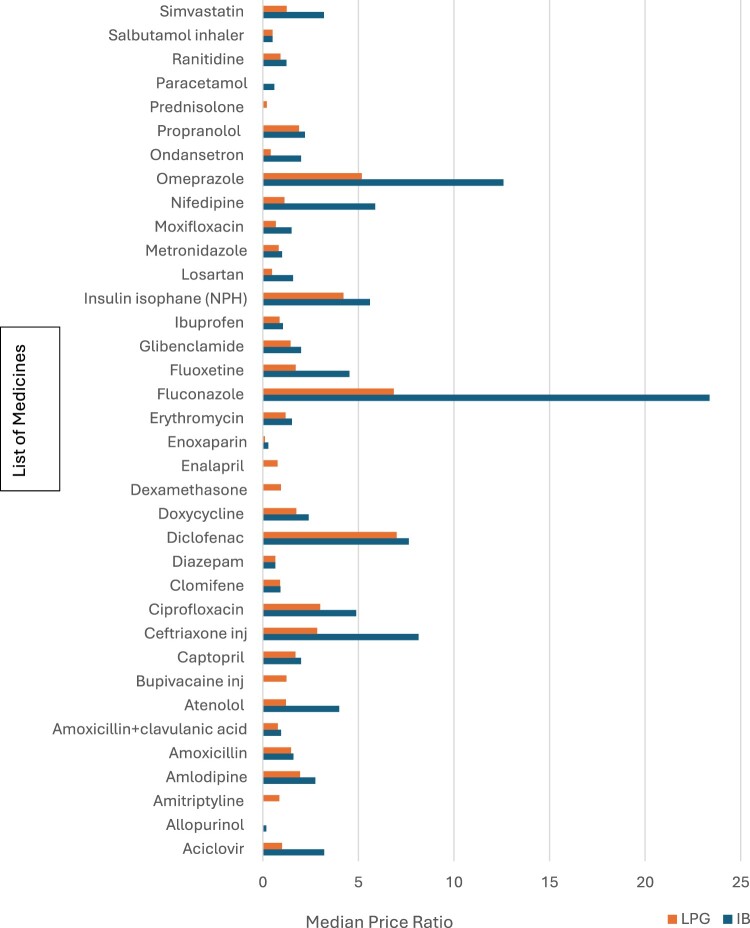
Median Price Ratios (MPR) for surveyed medicines in the private sector.

The data revealed that median price ratios for surveyed medicines within private retail pharmaceutical outlets varied between 0.11 and 23.7. Out of 50 medicines, the median price of 36 medicines was available in the international reference price MSH guide 2015. As prices were collected from the private sector, it was observed that the MPR of 18 LPG medicines was below 1, showing them cheaper in international comparison, and a total of 29 LPG (almost 80%) with less than 2 six IB had MPR > 1 but < 2,  and twelve LPGs were calculated with MPR of less than 2. 43.3% of innovator brands and 79.4% of lowest priced generic medicines were found with less than 2 MPR in private retail pharmaceutical outlets. The price variations for innovator brand (IB) were between 0.17 and 23.3. Overall, 33.3 per cent of medicines had MPR greater than 3, including Fluconazole (23.37), Ceftriaxone injection (8.15), Omeprazole (12.5), Diclofenac (7.64), and Insulin Isophane (5.6). Over all, 79.4% of LPGs were found with less than 2 MPR. Only twenty per cent of LPGs, including Diclofenac (7), Fluconazole (6.85), Nifedipine (5.88), Omeprazole (5.18), and Insulin Isophane (4.22), were found with greater than 2 MPR. Originator vs. Lowest-priced generic median price ratios are shown in Figure 1.

### Availability

Data for the present investigations were collected from pharmacies of 9 government hospitals and 18 private pharmacy outlets. The overall status of supply varied across sectors. In the private sector, the mean level of availability (in per cent) of the innovator brand is 45.7%, whereas in the public sector, it is only 12.65%. The mean level of availability of lowest priced generics (LPGs) was 36.2% in the public sector and 55.2% in the private sector. In the public sector, 8% of medicines showed more than 80% availability, and 24% of LPGs and 52% of IB were absent. Within the private sector, 12% of LPGs and 20% of IB were absent. Prazosin 1 mg tablet (LPG or IB) was not present in any of the sectors. A summary of the mean percent availability of all medicines, distinguishing between innovator products and lowest – priced generics in both sectors, is presented in Table 1.

**Table 1.** Individual medicine availability of IB & LPG in public and private sector.

Individual medicines availability in public and private pharmacies					
Medicine name & dosage form	Strength	Innovator brand(%)		Lowest priced generic(%)	
		Public (n = 9)	Private (n = 18)	Public (n = 9)	Private (n = 18)
Acyclovir tab	200 mg	11.1%	27.8%	44.4%	72.2%
Anastrozole tab	1 mg	0.0%	50.0%	11.1%	5.55%
Allopurinol tab	100 mg	44.4%	77.8%	0.0%	0.0%
Amitriptyline tab	25 mg	22.2%	38.9%	0.0%	50.0%
Amlodipine tab	5 mg	22.2%	61.1%	66.6%	77.8%
Amoxicillin cap	500 mg	22.2%	83.3%	55.5%	50.0%
Amoxicillin + clavulanic acid tab	875 + 125 mg	33.3%	61.1%	44.4%	38.9%
Atenolol tab	50 mg	44.4%	83.3%	33.3%	72.2%
Bupivacaine inj	2 ml	0.0%	0.0%	33.3%	44.4%
Captopril tab	25 mg	44.4%	83.3%	22.2%	44.4%
Ceftriaxone inj	1 gm	0.0%	83.3%	100.0%	66.7%
Ciprofloxacin tab	500 mg	0.0%	66.7%	100.0%	83.3%
Co-trimoxazole susp	8 + 40 mg	22.2%	55.0%	0.0%	0.0%
Clomifene tab	50 mg	22.2%	66.7%	11.1%	66.7%
Diazepam tab	5 mg	33.3%	38.9%	22.2%	50.0%
Diclofenac tab	50 mg	22.2%	66.7%	77.7%	50.0%
Doxycycline tab	100 mg	11.1%	61.1%	22.2%	83.3%
Dexamethasone tab	0.5 mg	0.0%	0.0%	55.5%	66.7%
Enalapril tab	10 mg	0.0%	0.0%	33.3%	66.7%
Enoxaparin inj	40 mg	22.2%	66.1%	22.2%	22.2%
Erythromycin cap	250 mg	22.2%	94.4%	0.0%	24.2%
Fluconazole tab	150 mg	0.0%	50.0%	11.1%	100%
Fluoxetine tab	20 mg	0.0%	88.9%	22.2%	72.2%
Gentimicin sulphate inj	80 mg	0.0%	0.0%	44.4%	66.7%
Glibenclamide tab	5 mg	55.5%	88.9%	11.1%	44.4%
Gliclazide tab	30 mg	11.1%	94.4%	22.2%	38.9%
Ibuprofen tab	400 mg	22.2%	22.2%	55.5%	66.7%
Irinotecan inj	20 mg/ml	0.0%	0.0%	0.0%	11.1%
Insulin isophane (NPH) vial	100IU/ml	0.0%	72.2%	77.7%	44.4%
Imatinib tab	400mg	0.0%	5.6%	0.0%	22.2%
Itraconazole tab	100 mg	0.0%	66.7%	11.1%	88.9%
Losartan tab	50 mg	0.0%	22.2%	33.3%	88.9%
Linezolid inj	600 mg/200ml	0.0%	0.0%	44.4%	38.9%
Metronidazole tab	400 mg	33.3%	88.9%	77.7%	55.5%
Montelukast tab	10 mg	0.0%	5.5%	55.5%	94.4%
Moxifloxacin tab	400 mg	0.0%	50.0%	44.4%	94.4%
Nifedipine tab	20 mg	0.0%	22.2%	0.0%	38.9%
Nystatin oral drops	50 ml	0.0%	0.0%	11.1%	83.3%
Ofloxacin tab	400 mg	0.0%	0.0%	0.0%	83.3%
Omeprazole cap	20 mg	0.0%	38.8%	100.0%	94.4%
Ondansetron tab	4mg	0.0%	38.8%	55.5%	77.8%
Prazosin tab	1 mg	0.0%	0.0%	0.0%	0.0%
Propranolol tab	40mg	44.4%	88.9%	0.0%	27.8%
Prednisolone tab	5mg	22.2%	50.0%	0.0%	38.8%
Paracetamol susp	24 mg/ml	22.2%	83.3%	77.8%	11.1%
Ranitidine tab	150 mg	0.0%	22.2%	22.2%	22.2%
Salbutamol inhaler	100 mcg/dose	22.2%	50.0%	33.3%	66.7%
Simvastatin tab	20 mg	0.0%	22.2%	11.1%	50.0%
Sofosbuvir cap	400 mg	0.0%	16.7%	22.2%	44.4%
Tranexamic Acid tab	500 mg	0.0%	0.0%	66.6%	88.9%

Source: Author’s calculations.

**Figure 2.**
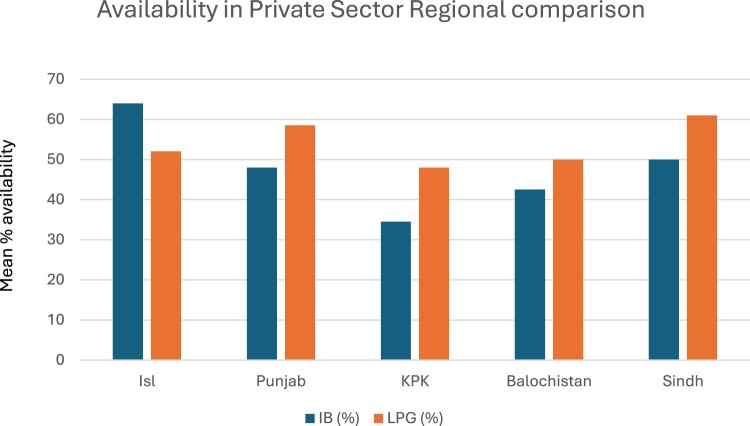
Availability in private sector a regional comparison.

In private sector retail pharmacies, results indicate that the Islamabad Capital Territory has a high availability of innovator brands at 64%, and Sindh showed a greater reliance on the lowest-priced generics at 61%, followed by Punjab at 58.5%. Khyber Pakhtunkhwa presents the lowest availability of 34.5% (IB) and 48% (LPG), while Balochistan reflects moderate access. The Islamabad private sector trend towards IB shows clear regional disparities. The regional comparison of availability is shown in Figure 2.

**Figure 3.**
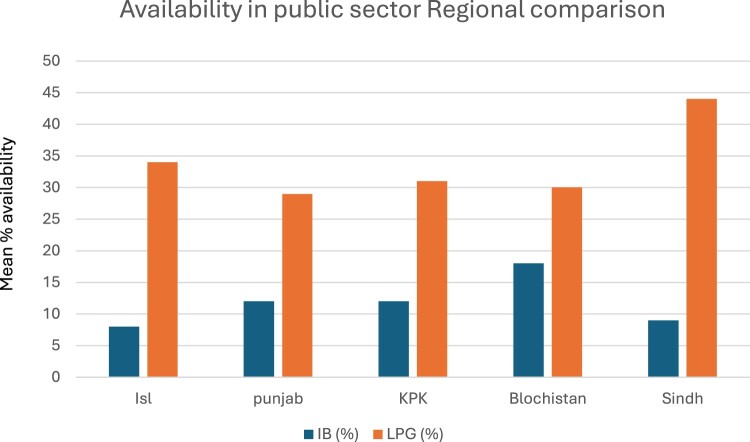
Availability in public sector a regional comparison.

Public sector data for Islamabad are higher than the private sector data, with just 8% availability of IB; however, 34% of LPGs were available. Balochistan showed surprisingly high innovator brand availability of 18%, and in Sindh, LPG use is quite satisfactory. Punjab and KPK indicate almost identical results. Results are shown in Figure 3.

### Affordability

As medicines are dispensed at no cost in public sector hospital pharmacies, the affordability was assessed based on the lowest wage of an unskilled government worker. This was calculated using private sector retail pharmacy prices. Treatment with Originator brands of simvastatin (1.45), Omeprazole (1.52), Ceftriaxone injection (6.5), and Fluconazole (15.08) exceeded a single day’s earning, making them unaffordable. The therapy with generic equivalents Ceftriaxone injection (2.27) and Fluconazole (4.37) was also unaffordable. Overall, an average of 5.6 days' wage was required for hepatitis C treatment with the lowest-priced generic and 21 days' wage with OB. If calculated for a full course of treatment, the average increased to 33.8 and 126.5 days' wage for LPG and OB, respectively. Affordability calculation for standard treatment by the lowest-paid government worker is described in Table 2.

**Table 2.** Affordability of Standard Treatment in private sector retail pharmacies.

Serial	Disease/condition	Medicine	Strength	Dosage form	No. of units as treated by	Duration of days	Median treatment price in Pakistani rupees		Day′s wage for treatment	
							IB	LPG	IB	LPG
1	Cardiovascular	Captopril	25 mg	tab	60	30	1161	969.6	0.87	0.72
		Amlodipine	5 mg	tab	30	30	501	355.8	0.37	0.26
		Simvastatin	20 mg	tab	30	30	1935	750	1.45	0.56
2	Asthma	Salbutamol	100 mcg/dose	inhaler	200	30	376	362	0.30	0.29
3	Diabetes	Insulin Isophane(NPH)	100IU/ml	Vial	10ml	30	1196	900	0.89	0.67
		Glibenclaimide	5 mg	tab	90	30	396.6	284.4	0.29	0.21
4	Ulcer	Omeprazole	20 mg	cap	30	30	2035	840	1.52	0.63
		Ranitidine	150 mg	tab	30	30	330	243	0.24	0.18
5.	Bacterial infection	Ceftriaxone	1g	inj	7	7	9708	3034	6.5	2.27
6.	Infertility	Clomiphene	50 mg	tab	20	10 days	1256	1235	1.01	1
7.	Fungal Infection	Fluconazole	150 mg	cap	30	15 days	18600	5400	15.08	4.37
8.	Hepatitis C	Sofosbuvir	400 mg	Tab	28	28 days	26000	6944	21.08	5.6
					24 weeks	Full course	156000	41664	126.5	33.8
9.	Depression	Fluoxetine	20 mg	cap	30	30	2250	855	1.82	0.7

Source: Author’s calculation.

## Discussion

The current study represents the comparison of IB and LPG in three distinct ways. First, some of the medicines, such as Salbutamol inhaler and Propranolol, showed almost identical prices for both LPG and IB. Second, medicines including Captopril, Diclofenac sodium, and Glibenclamide exhibited slight price difference between innovator brand and generic substitute. Strong regulatory oversight is essential to prevent generics from leveraging innovator prices to justify excessively high prices (Merchant et al., 2022). Current positive indicators of lower MPRs in the case of LPGs can be the result of Pakistan’s pharmaceutical industry’s shift to local firms, which hold 74.51% market share (Khan, 2024). Finally, the remaining LPGs were consistently priced lower than their innovator brand.

The previous studies has shown an average of 4–5 times lower to 25–60 times higher MPRs (Saeed et al., 2019) while the current study showed prices as low as 17 times and as high as 23.37 times. In all these studies, prices are compared based on MSH reference prices. Results showed that 43.75% of IBs out of available brands were overpriced, having an MPR of more than 2, and 20.58% of LPGs out of available generics were overpriced.

Compared with the 2019 study based on the survey data for 2016 and another study of Gujranwala district, the median price ratio of many innovator brands has declined in the current study. Acyclovir 20.21–3.21 for IB and LPG came down to 1.46 from 3.58 (2019); 4.64 (2022). IB of Amlodipine is now 2.75 as compared to 7.28; 6.82(2022), LPG of Amlodipine was 1.10 (2019); 3.65 (2022) and 1.95 in the current study. Ceftriaxone injection IB was 16.28 (2109); 15.68 (2022) came down to 8.15, Fluconazole 60.63 (2019); 57.60 (2022) to 23.37, Fluoxetine 9.71 (2019); 9.21 (2022) to 4.53, Omeprazole 34.04 (2019); 30.99 (2022) to 12.59, simvastatin 12.33 (2019); 11.71 (2022) to 3.2. MPR of LPG of Omeprazole decreased from 10.50 (2019); 6.81 (2022) to 5.18; Losartan 0.92–0.48. (Saeed et al., 2019; 2022).

However, some medicines showed an increase in MPRs, such as Nifedipine retard, which increased from 2.70–5.88 for IB. Propranolol's price increased from 2.12–2.23 for IB and 0.92–1.89 for LPG. Insulin isophane MPR increased from 1.12–5.6 for IB and from 0.83–4.22 for LPG.

These findings of the current study have shown consistently lower MPRs for both LPG and OB when compared to the Southeast Asian reviewed data in a secondary study by Lachlan published in The Lancet in 2025. For Fluoxetine, values ranged between 3.0 and 4.0, while the current study recorded 1.72. Similarly, the IB is 4.53 in Pakistan compared to 16.5 regionally. Salbutamol had the lowest MPRs close to international reference prices at 0.96–1 for LPG and 2.1 for IB, while the current data showed an even lower 0.51 for both. Ciprofloxacin exhibited a high IB of 27.5 and LPG around 3.9, compared to Pakistan’s 4.89 and 3.0, respectively. Glibenclamide in Southeast Asia was near 4.1 for LPG and approximately 20 for IB, but the current study recorded substantially reduced levels of 1.45 and 2, respectively. Taken together, these findings underscore that the current study in Pakistan demonstrates consistently lower MPRs for both LPG and IB when compared with the Southeast Asian data presented in Oldfield’s secondary analysis. These data are based on a multi-country study published in 2025, covering 54 countries (Oldfield et al., 2025). If compared to a WHO study in Bangladesh in 2019, the median price ratios of LPG and OB are lower in Pakistan, as Salbutamol is 1.4 and 1.2, but 0.51 for both in this study. IB Diclofenac in Bangladesh is 16.1 as compared to 7.64, but LPG is quite high in the current analysis at 7 compared to 1.7 in Bangladesh; however, metronidazole was 1.9 for OB and 1.5 for LPG in Bangladesh, and 1 and 0.83 in the current results (Kasonde et al., 2019). Although showing lower ratios in comparison, 50% (MPR>1) of the LPG and 28% (MPR>3) of the IB medicines are overpriced in relation to MPRs.

The outcomes of the current investigation regarding availability are in accordance with the findings that availability in Pakistan remained significantly below the WHO target of 80% by 2025, described in a five-year review of access to medicines in Pakistan (Malik et al., 2020). It was recorded in the current study that medicines used for cancer treatment were largely absent in the public sector and only sporadically accessible in private retail pharmacies. Availability of Irinotecan LPG (11.1%), Imatinib IB (5.5%), LPG (22.2%), and Anastrozole IB (5.5%) was extremely low. Most retail pharmacies indicated that expensive cancer medicines were supplied only upon specific demand, highlighting significant gaps in accessibility. In Pakistan, more people die after diagnosis due to cancer as compared to developed countries (Bhojwani et al., 2026), who have been implementing international strategies to increase access to high-priced pharmaceuticals (Hasan et al., 2018). In the present study, the mean availability of IB and LPG demonstrated considerable variation across sectors. These figures contrast with findings from national studies, such as a national survey in Pakistan that showed improvements of 28.8% for IB and 30.7% for LPG (Shahnaz, 2014), over the first survey of 2006 with OB at 0% and LPG at 3.3% (Kiani et al., 2006), and other regional studies such as Lahore at 6.8% and 35.3% (Saeed et al., 2019), Balochistan at 9.8% and 49.4% (Bibi et al., 2022), and Gujranwala at 7.9% and 8% (Shahzad et al., 2022). In the private sector, availability was higher with IB at 45.7% and LPG at 52.6%, while Lahore reported 55% and 20.3%, Balochistan at 51.8% and 42.6%, and Gujranwala at 68.5% and 78.7% for IB and LPG, respectively. A comprehensive document analysis also revealed persistent gaps in the regular supply of life-saving drugs (Rafi et al., 2021). All these findings are still aligned with a global study in 2013 on asthma medicines across 52 LMICs, which revealed profound disparities in the availability and cost of vital treatments (Babar, Lessing, et al., 2013). However, nowadays, strong generic competition has made the availability situation much better. They may determine their pricing based on production costs and maintain only minimal profit margins (Khoso et al., 2014).

When compared internationally, IB in the current results was higher than availability in Iran, where it was 6.4% in the public facilities and 8.6% in the private retail pharmacies. However, LPG and IB availability in the public sector was lower in the present study by 44.7% Compared to 45.4% and 75.5% in Iran (Ghanbari et al., 2024). A study conducted in China reported IB availability ranging from 4.29% to 9.27% in the government sector, which is less than current findings, and 13.50% to 43.75% in the private sector. In contrast, LPG availability in China was reported at 11.75% to 32.87% in the public sector and 26.29% to 39.54% in the private sector (Yang et al., 2020), again lower than the present study. Vietnam demonstrated prominently low IB availability of 13.7% in the private health sector and 0.7% in the government sector (Nguyen et al., 2021). The current results showed IB availability was more than that of Ethiopia at 1.43% and 5.50%, and LPGs at 42.5% and 50.8% for the public and private sectors, respectively (Sisay et al., 2021). It was also observed that many of the public sector hospitals had the essential medicines in the form of injections.

Affordability of LPG versions, when compared to the African region, is quite affordable in Pakistan; Diazepam, which needed more than 2 days' wage for generic treatment, is calculated with a median price ratio of less than 1, henc, it is easily affordable. Salbutamol inhaler required 15 days' wage in Juba County and requires half a day’s wage in Pakistan. Omeprazole requires 0.63 day' wage with LPG in comparison to 4 day s' wage in Juba (Deng et al., 2023). However, the treatment for Hepatitis C is extremely unaffordable, even if treated with LPGs. Although the nominal wages have increased 62% from 2020–2021 to 2025-2026, inflation, poor social protection, limited security, and the informal labour market (Abbasi Kasim, 2026) constrained the ability to improve health and lifestyle.

## Limitations

This research assesses the availability and pricing of medicines in major cities across each province. However, availability may vary at primary and secondary health care levels in far-off areas. Some medicines were identified in strengths different from those specified in the survey form, and 16 medicines' supplier MPRs were not found in the MSH guide; therefore, their availability and median price ratios comparison was not included in the analysis. Affordability calculations, based on the government minimum daily wage, may overestimate actual affordability since many private sector employees earn less, and multiple patients having multiple chronic diseases may depend on a single household income.

## Conclusion

Overall, the prices of the lowest-priced generics decreased for most of the medicines. However, the prices of some medicines also increased (when compared with median price ratios relative to the international reference price). It was observed that approximately half of the innovator brands were highlypriced. No regional variations were observed regarding the prices of innovator brands and generics. Essential Medicines remained insufficiently available in the public sector. It was also observed that more than half of the WHO core list medicines were affordable. It was noted that medicines used for diabetes, cancer, hepatitis, and infectious diseases were unaffordable. Over the years, the availability of generic medicines has improved, especially in the private sector.

## Recommendations

There must be a uniform, transparent, and sustainable procurement and supply chain management system designed at the federal level to ensure the regular availability of medicines across all public hospitals. A separate authority needs to be established for price setting and monitoring of medicines. Furthermore, coordinated, evidence-based and targeted policy actions are needed to address economic and logistical barriers, including inefficiencies in the medicines distribution system.
